# Analysis of the Enzymatic Activity of an NS3 Helicase Genotype 3a Variant Sequence Obtained from a Relapse Patient

**DOI:** 10.1371/journal.pone.0144638

**Published:** 2015-12-10

**Authors:** Paola J. S. Provazzi, Sourav Mukherjee, Alicia M. Hanson, Mauricio L. Nogueira, Bruno M. Carneiro, David N. Frick, Paula Rahal

**Affiliations:** 1 São Paulo State University - UNESP, Department of Biology, São José do Rio Preto/SP, CEP: 15054–000, Brazil; 2 University of Wisconsin- Milwaukee, Department of Chemistry & Biochemistry, Milwaukee, WI, 53217, United States of America; 3 São José do Rio Preto Medical School, Laboratory of Virology, São José do Rio Preto/SP, CEP: 15090–000, Brazil; Centro de Biología Molecular Severo Ochoa (CSIC-UAM), SPAIN

## Abstract

The hepatitis C virus (HCV) is a species of diverse genotypes that infect over 170 million people worldwide, causing chronic inflammation, cirrhosis and hepatocellular carcinoma. HCV genotype 3a is common in Brazil, and it is associated with a relatively poor response to current direct-acting antiviral therapies. The HCV NS3 protein cleaves part of the HCV polyprotein, and cellular antiviral proteins. It is therefore the target of several HCV drugs. In addition to its protease activity, NS3 is also an RNA helicase. Previously, HCV present in a relapse patient was found to harbor a mutation known to be lethal to HCV genotype 1b. The point mutation encodes the amino acid substitution W501R in the helicase RNA binding site. To examine how the W501R substitution affects NS3 helicase activity in a genotype 3a background, wild type and W501R genotype 3a NS3 alleles were sub-cloned, expressed in *E*. *coli*, and the recombinant proteins were purified and characterized. The impact of the W501R allele on genotype 2a and 3a subgenomic replicons was also analyzed. Assays monitoring helicase-catalyzed DNA and RNA unwinding revealed that the catalytic efficiency of wild type genotype 3a NS3 helicase was more than 600 times greater than the W501R protein. Other assays revealed that the W501R protein bound DNA less than 2 times weaker than wild type, and both proteins hydrolyzed ATP at similar rates. In Huh7.5 cells, both genotype 2a and 3a subgenomic HCV replicons harboring the W501R allele showed a severe defect in replication. Since the W501R allele is carried as a minor variant, its replication would therefore need to be attributed to the *trans*-complementation by other wild type quasispecies.

## Introduction

The hepatitis C virus (HCV) infects at least 170 million people in the world, causing acute and chronic hepatitis, liver cirrhosis and hepatocellular carcinoma [1–4]. HCV is a positive sense single-stranded RNA virus with a ~9,600 nucleotide genome that encodes a single ~3,000 amino acid polyprotein [5–7]. Host and cellular proteases cleave the HCV polyprotein into the HCV structural proteins (core, E1, and E2) and nonstructural proteins (p7, NS2, NS3, NS4A, NS4B, NS5A, and NS5B) [8]. Until recently, all HCV treatments were based on the combination of pegylated interferon, ribavirin, and NS3 protease inhibitors, but all-oral interferon-free direct acting antiviral (DAA) combinations are now the standard of HCV care. The HCV protease inhibitors telaprevir (Vertex Pharmaceuticals) and boceprevir (Merck & Co) [9] were the first HCV DAAs, and they target the NS3/NS4A serine protease [10]. These early drugs have now been replaced by other DAA combinations that use a variety of NS5B, NS5A and NS3 inhibitors. The new combinations cure most patients, but HCV patients with genotype 3a respond less well to the new drugs than patients infected with other genotypes [11,12].

After self-cleavage, the NS3/NS4A serine protease is a hetero-dimer, in which the catalytic triad resides in the N-terminal 181-residue “protease” domain of the 631-residue NS3 protein. Residues 21 to 30 of the 54-residue NS4A protein bind NS3 to activate the protease [13–16]. The NS3/4A protease cleaves the NS3/NS4A, NS4A/NS4B, NS4B/NS5A and NS5A/NS5B junctions in the HCV polyprotein and also cellular proteins needed for antiviral signaling [17–21]. In addition to its protease function, NS3 is also a helicase essential for HCV replication [22,23]. The NS3 helicase activity resides in the C-terminal NS3 domain, which also has nucleic acid-stimulated (d) NTPase activity [24]. The NS3 helicase is a superfamily-2 (SF2) DExH/D-box helicase that unwinds both RNA and DNA in a 3′-5′ direction [25,26]. Truncated NS3 lacking the protease domain (NS3h) can be expressed in *E*. *coli* and purified as an active helicase. NS3h has three domains [27,28]. Domain 1 contains the Walker A (^264^AXXXXGKS^211^) and B (^290^DEXH^293^) NTP-binding motifs. Domain 2 contains a conserved Arg-rich motif (^460^QRRGRTGR^467^) also needed for ATP hydrolysis and is the second motor domain conserved with other superfamily 1 & 2 helicases. Domain 3 has a predominantly alpha helical structure and is not shared with related SF2 helicases [29]. DNA binds in a groove between domain 3 and the two motor domains, with the 3’ terminal base stacked against Trp501 in domain 3. Trp501 acts as a bookend to define the border of the substrate-binding cavity. The residue moves when ATP binds and is hydrolyzed to allow translocation of the helicase in the 3′-5′ direction [27,30]. Potential antivirals that inhibit the HCV helicase have been identified [31–33], and some of these drug candidates bind near W501 [34].

The natural occurrence of the amino acid substitution W501R in NS3 was previously reported in a patient infected with HCV genotype 3a who responded to interferon/ribavirin therapy, but in whom virus levels rebounded after treatment was ended [35]. In HCV genotype 1, substitution of Trp501 with a non-aromatic amino acid has been previously shown to lead to poor NS3-catalyzed nucleic acid unwinding [29] and block genotype 1b replication in cells [23]. However, because all prior studies were performed with HCV genotype 1a or 1b, we analyzed the impact of the W501R substitution in a genotype 3a background to test the hypothesis that other residues that differ between genotypes 1b and 3a might compensate for this defect. Our results showed that wild type genotype 3a helicase is more active than the genotype 1b helicase, and that a genotype 3a helicase with the W501R substitution unwinds DNA or RNA poorly, even though it retains an ability to bind nucleic acid and hydrolyze ATP. In a genotype 3a replicon the W501R allele again blocked replication.

## Experimental Procedures

### Ethics Statement

The project was approved by the research ethics committee of the São José do Rio Preto School of Medicine (FAMERP; opinion Nr. 087/2004), and all participants signed an informed consent.

### Population and Samples

The study material consisted of serum samples obtained from16 patients infected with HCV 3a. The mean age at the time of diagnosis was 47.7 years old. After confirming the positive diagnosis of infection, which was defined by positivity for the virus antibody through ELISA and qualitative PCR for HCV RNA, the patients were given 24 weeks of treatment with interferon-alpha and ribavirin, and were followed up for up to 6 months after treatment. Serum collections were performed at 12 and 24 weeks during treatment, 7, 14, 21 and 28 days after the treatment had been completed, and monthly thereafter for 6 months. No patients were co-infected with the human immunodeficiency virus or with the hepatitis B virus.

### Extraction of RNA and Amplification of the NS3 Helicase region (NS3h)

Viral RNA was extracted from blood serum samples obtained 6-months after treatment was ended using a QIAamp Viral RNA Mini Kit (Qiagen), and cDNA was synthesized using a High-Capacity cDNA Archive Kit (Applied Biosystems by Life Technologies). The cDNA was amplified using the NS3-specific primers (forward 5'- GGAATTCCATATGTCCCCATCTTTCTCTGACAATTCAACT-3' with underlined NdeI site, and reverse 5'-CGCGGATCCTCAGGTGGTTACTTCCAGATC-3', with underlined BamHI site) for the helicase domain of NS3 (NS3h) (181–631 aa). Primers used to sequence cloned cDNA were: Seq787F 5' GCCAAA CTGACCTATTCCAC 3'; Seq980F 5' AGCATCACTGTGCCACATTC 3'; Seq1216F 5' GTCGTAGTTTGCGCTACTG 3'; Seq1654R 5' GCTTAGTCTGTGACAGAAAGTG 3'; Seq1454R 5' ATT CCAGACGGTCTTTCACC 3'; Seq1005R 5' GTTAGAATGTGGCACAGTGATG 3'; M13 forward 5' GTAAAACGACGGCCAG 3'; M13 reverse 5′ CAGGAAACAGCTATGAC 3′). PCR was performed in 20μl with100 to 500 ng of cDNA in 1.6x buffer, 200 μM of each dNTP, 4 mM MgCl_2_, 0.5 mM primers, and 5 units of Elongase^®^ Enzyme polymerase (Invitrogen by Life Technologies, Grand Island, NY, USA). Cycling consisted of an initial step of 2 min at 94°C, 40 cycles of 1 min at 94°C, 1 min for primer annealing at a temperature of 55°C, and 3 min at 72°C for extension of the chains. The final extension time was 15 min at 72°C. The resulting amplicon was 1,350 bp.

### Cloning

To search for sequence mutations, the PCR amplicons were cloned in the PCR-XL-TOP cloning vector (Invitrogen), using the Topo XL PCR Cloning Kit (Invitrogen). Ligation products containing inserts were identified by amplifying the cloning region using the M13 forward and reverse primers in a PCR. Fifteen cDNA clones from each patient were selected for sequencing [36–39]. Each was grown in 3.5 ml of LB containing 50 μg/ml kanamycin, and each plasmid was isolated using the SNAP Miniprep Kit (Invitrogen).

For protein expression, the NS3h regions of wild type and W501R NS3 were subcloned into a T7 expression vector. Both XL TOPO cloning vectors and pET28a (Novagen) were cleaved with NdeI and BamHI. The purified DNA fragments were ligated with T4 DNA ligase (Fermentas). After plasmids with appropriate sequences inserted downstream of the T7 promoter in frame with the His-tag were identified using DNA sequencing, the purified plasmids were used to transform BL21(DE3) cells for protein expression and purification (see below).

### Protein purification

All recombinant NS3helicase proteins analyzed in this study (NS3h_1b (con1), NS3h_3a (wt) and NS3h_3a (W501R)) contained NS3 amino acids 181 to 631 fused to a C-terminal hexa-histidine tag that was not removed before analysis. NS3h_3a (W501R) was expressed from the plasmid JES/pET28a, and the NS3h_3a (wt) was expressed from the plasmid TUG/pET28a. The recombinant His-tagged proteins were expressed and purified from *Escherichia coli* BL21 (DE3) that had been transformed with one of the aforementioned plasmids. The recombinant proteins were purified as described previously [40]. Briefly, all proteins were expressed by adding Isopropyl β-D-1-thiogalactopyranoside to 1 L cultures when the OD_600_ was about 1.0, and growing the cultures for 2–3 additional hours at 23°C. Proteins were purified using metal affinity, gel filtration, and ion-exchange chromatography and ammonium sulfate fractionation exactly as described previously [40]. After dialyzing the purified protein into 20 mM Tris, pH 8, 50 mMNaCl, 1 mM EDTA, 0.1 mM DTT, 25% glycerol, concentrations were determined by measuring A_280_ using extinction coefficients determined with the program Sequence Analysis (Informagen, Greenland, NH).

### Nucleic Acid Unwinding (helicase) assays

A molecular beacon-based helicase assay (MBHA) was used to measure DNA unwinding, and a RNA-based split-beacon helicase assay (SBHA) was used to measure helicase-catalyzed RNA unwinding, as described [40]. Briefly, MBHAs were performed in 96-well microplates, at 23°C. A total volume of 60 μl was used, containing 25 mM MOPS (pH 6.5), 1.25 mM MgCl_2_, 0.05 mM DTT, 0.005 mg/mL BSA, 0.001% Tween20, 5 nM Cy-5-MBHA substrate, 1 mM ATP and various enzyme concentrations from 0 to 4,900 nM for the mutant enzyme and from 0 to 200 nM for NS3h_1b (con1) and NS3h_3a (wt). SBHAs were performed in 96-well white microplates in 60 μl with 25 mM MOPS (pH 6.5), 1.25 mM MgCl_2_, 0.05 mM DTT, 0.005 mg/mL BSA, 0.001% Tween20, 10 nM substrate, 1 mM ATP and various enzyme concentrations from 0 to 4,900 nM for the mutant enzyme and from 0 to 200 nM for wild type enzymes. Data were analyzed with Graphpad Prism (La Jolla, CA, USA) as described [40].

### DNA Binding Assays

A fluorescence polarization (FP)-based DNA binding assay was used to estimate the affinity of each protein for DNA as described [32]. Reactions were performed in 384-well microplates in 20 μl with 25mM MOPS (pH 6.5), 1.25 mM MgCl_2_, 5 nM Cy5-dT15, 0.05 mM DTT, 0.005 mg/mL BSA, 0.001% Tween20, and various concentrations of each enzyme. Polarization was monitored with a TECAN Infinite M1000 PRO multi-mode microplate reader and the data were analyzed with Graphpad Prism (La Jolla, CA, USA).

### ATPase Assays

The NS3 protein’s ability to hydrolyze ATP was determined using a colorimetric assay as described before [32]. Reactions were prepared in duplicate in 96-well plates, in a total volume of 30 μl containing 25 mM MOPS (pH 6.5), 1.25 mM MgCl_2_, 1 mM ATP, 1 nM, 0.05 mM DTT, 0.005 mg/mL BSA, 0.001% Tween20, of each enzyme and 5.0 μl of one of 12 serial dilutions from 0 μM to 10 μM of poly (U) and poly (A) RNA. The ATPase reaction was terminated malachite green reagent and sodium citrate. The absorbance was read at A_630_ and converted to phosphate using a standard curve generated from mock reactions containing only inorganic phosphate. Data were analyzed with Graphpad Prism (La Jolla, CA, USA) using the Michaelis-Menten equation.

### HCV Replicon Assays

The genotype 2a replicon reporter plasmids pSGR-Luc-JFH1 and pSGR-Luc-JFH1/GND [41] were generous gifts from John McLaughlin (MRC Virology Unit, Institute of Virology, Glasgow, United Kingdom). The genotype 3a replicon plasmid pS52/SG-Feo(AII) [42] was a kind gift of Dr. Charles Rice (Rockefeller University, New York, NY). To construct pSGR-Luc-W501R-JFH1, the plasmid pSGR-Luc-JFH1 was altered using the QuikChange II Site-Directed Mutagenesis Kit (Agilent Technologies, Santa Clara, CA, USA). The mutated fragment was digested with *NsiI* and *SpeI* restriction enzymes (Promega, Madison, WI, *USA)* and inserted using T4 DNA Ligase (Fisher Scientific, Pittsburgh PA, USA) into the pSGR-Luc-JFH1 wild type. The genotype 3a replicon containing the W501R mutation (pS52/SG-W501R-Feo) was constructed in a similar fashion. Each mutant allele was verified using DNA sequencing. The entire replicon open reading frame was sequenced, and no other mutations were detected.

Human hepatoma Huh 7.5 cells, provided by Apath LLC, Brooklyn, NY, were cultured in Dulbecco’s Modified Eagle’s medium (DMEM) (Gibco, Grand Island, NY, USA) and supplemented with 10% fetal bovine serum (Cultilab, Campinas, SP, Brazil), 1% nonessential amino acids (Gibco), 100 units/ml penicillin and 100 μg/ml streptomycin (Invitrogen by Life Technologies, Grand Island, NY, Grand Island, NY, USA) at 37°C in 5% CO_2_ atmosphere. The purified plasmid DNA was linearized by *XbaI* digestion (New England BioLabs, Ipswich MA, USA), treated with *MungBean* nuclease (New England BioLabs, Ipswich MA, USA) to remove overhangs, and transcribed using the MEGAscript T7 kit (Ambion, Austin, TX, USA). RNA was then isolated using *DNase I* (Ambion) and TRIzol (Invitrogen by Life Technologies, Grand Island, NY), and used to transfect cells as described [43]. For the assays with the genotype 2a replicons, 1x10^5^ cells were seeded in triplicates in 12-well culture plates, and maintained at 37°C in 5% CO_2_ for 4, 24, 48 and 72 h, when luciferase levels were used to estimate the HCV RNA levels. The cells were washed twice with phosphate-buffered saline and lysed with ice-cold passive lysis buffer (Promega, Madison, WI, USA). The cell lysate (25 μl) was mixed with 50 μl of the luciferase assay reagent (Promega) and luminescence was measured with a FLUOstar Omega multimode microplate reader (BMG LABTECH GmbH, Allmendgruen 8, Ortenberg/Germany). The readings at 4 h were used to normalize values obtained at later times. All experiments were performed twice, each time in triplicate.

Experiments with the genotype 3a replicons used more cells because this replicon is less robust than the genotype 2a (JFH1) replicon, and no reporter gene was present. About 2 x 10^6^ transfected cells were transferred to 10 cm² plates and 48 h after electroporation selection media containing 500μg/mL G418 (Sigma Aldrich) was added to the culture. The medium was changed every three days until it was no longer possible to observe dead cells in the supernatant. Colony containing plates were then fixed with 10% formaldehyde and stained with 0.01% (w/v) crystal violet. Images of the colonies were obtained using a scanner, and colonies were counted using the Zeiss Zen lite 2011 software (Zeiss).

### Statistical Analysis

For enzymatic assays the time courses collected fluorescence data were exported to the Graphpad Prism (La Jolla, CA, USA) and fitted to the Michaelis-Menten equation using the Prism software. The resulting data were compared statistically by the F test that determines whether the difference between the values is statistically significant (P< 0.0001). The curve fits were checked for all graphics.

For HCV replicon assays, all experiments were performed twice, each time in triplicate. Data were normalized to luciferase activity values at 4 h and bars represent the mean of two independent experiments with variability below 15%.

## Results

In a prior study, RNA encoding wild type HCV genotype 3a NS3 helicase and RNA with a mutant allele encoding NS3 helicase with a W501R substitution were isolated from patients being treated for HCV infection [35]. In that study, patient serum samples were subjected to RNA extraction, cDNA synthesis, PCR amplification, cloning and sequencing to generate 5 contig sequences of the helicase-coding regions for each patient. The NS3 W501R allele was isolated from patient RF020 [35]. Patient RF020 was diagnosed with HCV genotype 3a. The patient responded to interferon/ribavirin therapy, but relapsed three months after the end of treatment. Wild type genotype 3a NS3 was isolated from Patient RF009, who was also diagnosed with HCV genotype 3a, responded to therapy, and also relapsed. DNA amplified from patients was subcloned into a T7 expression vector and used to express wild type (NS3h_3a (wt)) and mutant (NS3h_3a (W501R)) helicases. Since NS3h_3a (wt) has not been extensively characterized before, both proteins were compared to the well-characterized NS3h isolated from the con1 strain of HCV genotype 1b (NS3h_1b (con1)).

### The W501R substitution profoundly influences NS3-catalyzed DNA and RNA unwinding

**Fig 1:**
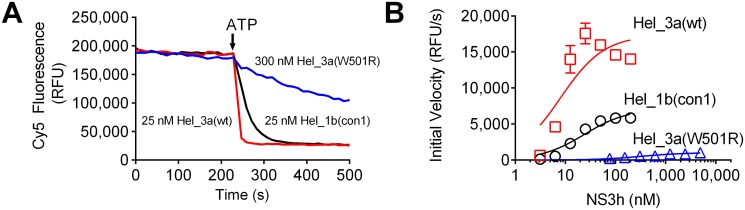
Effect of the W501R substitution on the ability of HCV helicase to unwind DNA. (A) Representative fluorescence traces observed in molecular beacon-based helicase assays **[36]** with indicated amounts of each protein. The decrease in fluorescence intensity (RFU—Relative Fluorescence Unit) indicates higher DNA unwinding activity of the wildtype enzymes than NS3h_3a (W501R). (B) Initial rates of unwinding after ATP addition in each reaction fit to the Michaelis-Menten equation. The reactions were prepared containing 25 mM MOPS, 1.25 mM MgCl_2_, 5 nM Cy5-substrate; 1mM ATP, 0.05 mM DTT, 0.005 mg/mL BSA, 0.001% Tween20, and enzymes NS3h_1b (con1), NS3h_3a (wt) and NS3h_3a (W501R) at indicated concentrations.

A molecular beacon-based helicase assay (MBHA) [44] was used to compare the relative abilities of the NS3h_3a (W501R), NS3h_3a (wt) and NS3h_1b (con1) proteins to catalyze DNA unwinding. The MBHA uses a dual-labeled hairpin-forming DNA oligonucleotide annealed to a longer oligonucleotide, which forms a tail for the helicase to load. Once ATP is added, the helicase displaces the molecular beacon, resulting in a decrease in substrate fluorescence. Eight different enzyme concentrations were used ranging from 0 nM to 40 nM for the wild type proteins and 0 to 4,900 nM for the mutant enzyme (Fig 1). The NS3h_3a (W501R) protein showed a reduced ability to unwind DNA compared to the NS3h_3a (wt) and NS3h_1b (con1) proteins. Interestingly, NS3h_3a (wt) was more active than NS3h_1b (con1). Some DNA unwinding was detected at very high protein concentrations of NS3h_3a (W501R), but rates were far lower than those observed with the wild type at far lower concentrations (Fig 1A). In the genotype 3a background, the W501R substitution affected both the apparent K_m_ and V_max_. As a consequence, the catalytic efficiency (V_max_/K_m_) of NS3h_3a (wt) was more than 600 times greater than the NS3h_3a (W501R) enzyme (Fig 1B and Table A in S1 File).

**Fig 2:**
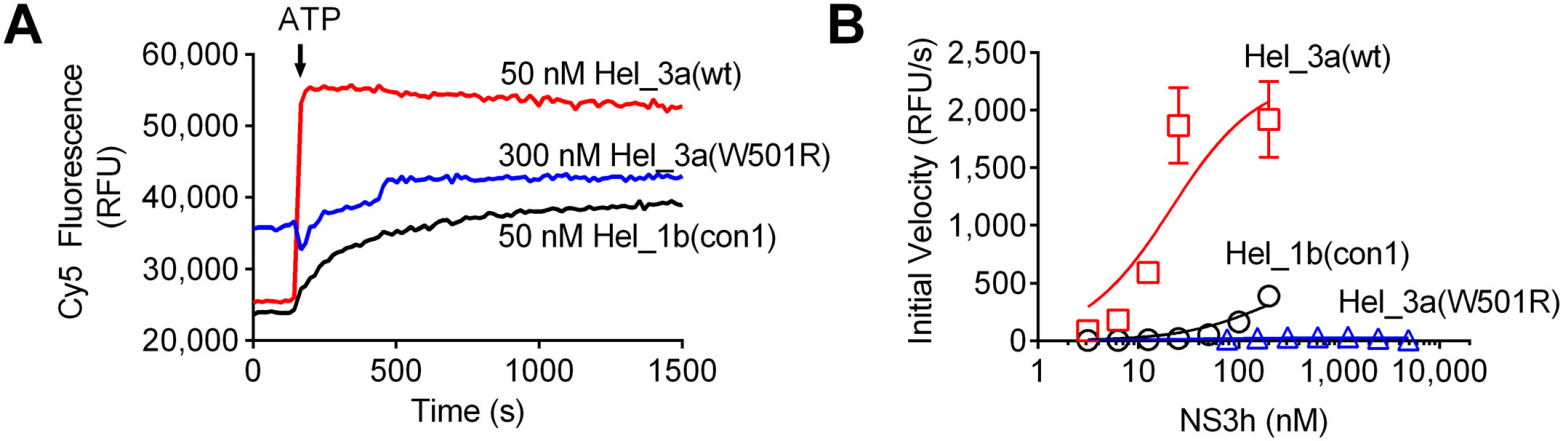
Effect of the W501R substitution on ability of HCV helicase to unwind RNA (A) Representative fluorescence traces observed in split beacon helicase assay **[36]** withindicated amounts of each protein. The increase in rates of fluorescence intensity (RFU—Relative Fluorescence Unit) change indicates higher activity of NS3h_3a (wt) and NS3h_1b (con1) enzymes in RNA unwinding compared to the mutant protein. (B) Initial rates of unwinding after ATP addition in each reaction fit to the Michaelis-Menten equation. The reactions were prepared containing 25 mM MOPS, 1.25 mM MgCl_2_, 5 nM Cy5, 0.05 mM DTT, 0.005 mg/mL BSA, 0.001% Tween20, substrate, 1mM ATP and enzymes NS3h_1b (con1), NS3h_3a (wt) and NS3h_3a (W501R) at indicated concentrations.

Tests were also performed using RNA as a substrate because RNA is the more likely natural substrate for NS3h (HCV has no DNA stage in its replication cycle). RNA-based assays where the fluorophore and quencher are split between two different hairpin forming oligonucleotides that both anneal to a third strand at adjacent positions were used [40]. In this split beacon helicase assay (SBHA), the two oligonucleotides that the helicase must separate for a signal change are made of RNA. Unlike in an MBHA, fluorescence increases when the helicase unwinds a SBHA substrate [40]. In SBHAs, NS3h_3a (wt) was about 3-times more active than NS3h_1b (con1), and unwinding was detected only with high concentrations of the NS3h_3a (W501R) protein (Fig 2A and Table A in S1 File). The catalytic efficiency of the mutant was about 100-times less than that observed with the wild type protein (Fig 2B and Table A in S1 File).

### The NS3 W501R protein retains an ability to bind DNA

**Fig 3:**
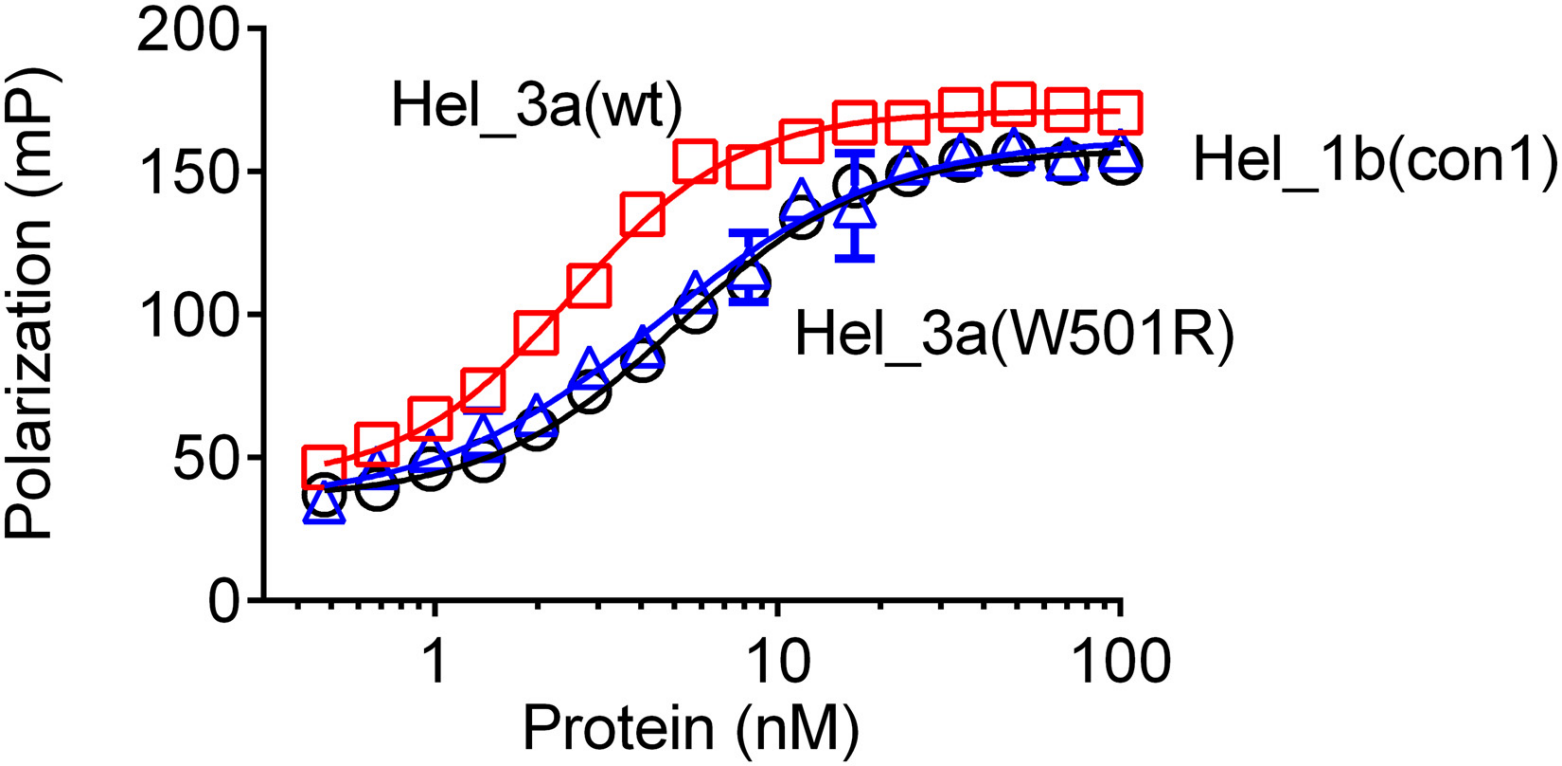
Effect of the W501R substitution on ability of HCV helicase to bind DNA. An increase in the polarization of Cy5-dT15 occurs when NS3h binds DNA. The reactions were prepared containing 25 mM MOPS (pH 6.5), 1.25 mM MgCl_2_, 100 nM Cy5, 0.05 mM DTT, 0.005 mg/mL BSA, 0.001% Tween20, and the enzymes NS3h_1b (con1), NS3h_3a (wt) and NS3h_3a (W501R) at indicated concentrations.

Direct DNA binding assays were next performed to test the hypothesis that the inability of NS3h_3a (W501R) to unwind DNA or RNA is due to an inability to bind nucleic acids. Specifically, a fluorescence polarization assay was used to detect the binding of a small fluorescently labeled oligonucleotide to the NS3h proteins [45]. The NS3h_3a (W501R) DNA binding was analyzed, and once again, NS3h_3a (wt) and NS3h_1b (con1) enzymes were evaluated for comparison. Binding assays were performed at enzyme concentrations ranging from 0 nM to 100 nM and analyzed as described before [32]. In binding assays, NS3h_3a (W501R) bound DNA only 1.7-times weaker than NS3h_3a (wt). NS3h_3a (W501R) and NS3h_1b (con1) bound with similar affinities (Fig 3 and Table A in S1 File).

### The NS3 W501R protein retains an ability to hydrolyze ATP

**Fig 4:**
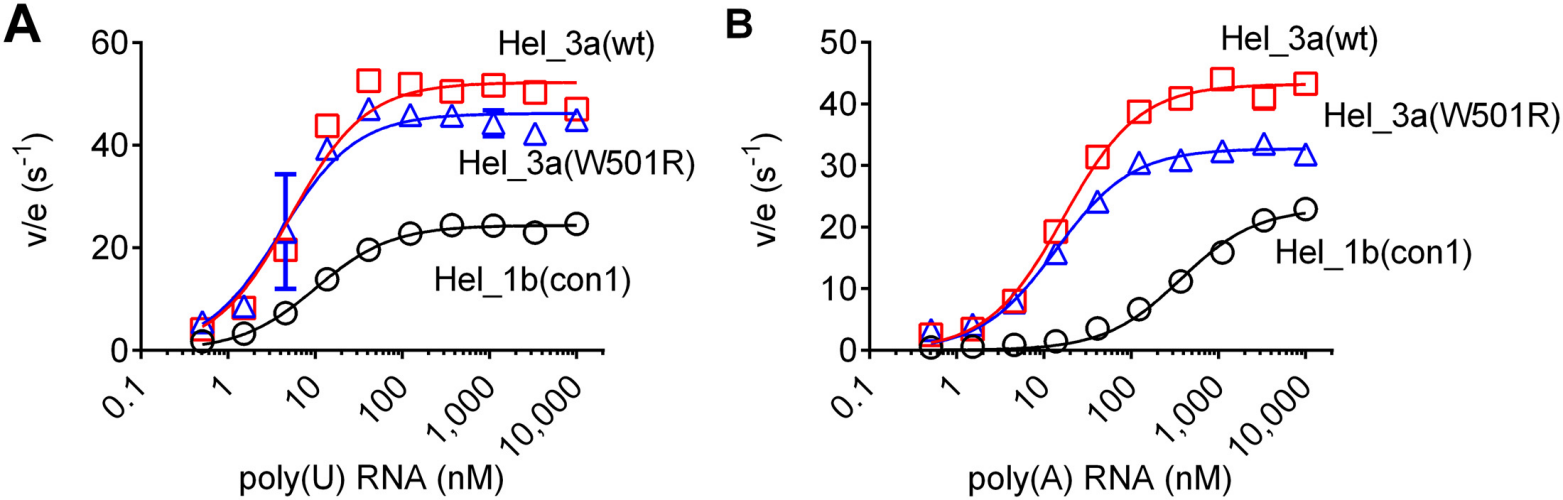
Effect of the W501R substitution on the ability of RNA to stimulate HCV helicase-catalyzed ATP hydrolysis. The increase in reaction rates (expressed as specific activities v/e (s^-1^)) indicates greater stimulation of the ATP hydrolysis activity. The reactions were prepared containing25 mM MOPS (pH 6.5), 1.25 mM MgCl_2_, 1 mM ATP, 1 nM, 0.05 mM DTT, 0.005 mg/mL BSA, 0.001% Tween20, and different concentrations of (A) poly (A) and (B) poly (U) RNA ranging from 0 nM to 10,000 nM.

To understand how the substitution affects the ability of RNA to stimulate helicase-catalyzed ATP hydrolysis and RNA binding, ATPase assays [46] were performed with each protein in the presence of various nucleic acid concentrations. Assays were performed with either poly (A) (Fig 4A) or poly (U) RNA (Fig 4B) as activators in concentrations ranging from 0.5 nM to 10,000 nM. Interestingly, both genotype 3a proteins hydrolyzed ATP with a faster V_max_, than the genotype 1b protein. However, the W501R substitution in NS3 helicase, did not alter either the apparent V_max_ or K_act_ for RNA observed with poly (U) RNA (Fig 4A and Table A in S1 File). The mutation only altered the apparent V_max_ and K_act_ observed with poly (A) RNA, but only slightly (33% and 21%, respectively) (Fig 4B and Table A in S1 File).

### Subgenomic HCV replicons do not tolerate an NS3 W501R allele

**Fig 5:**
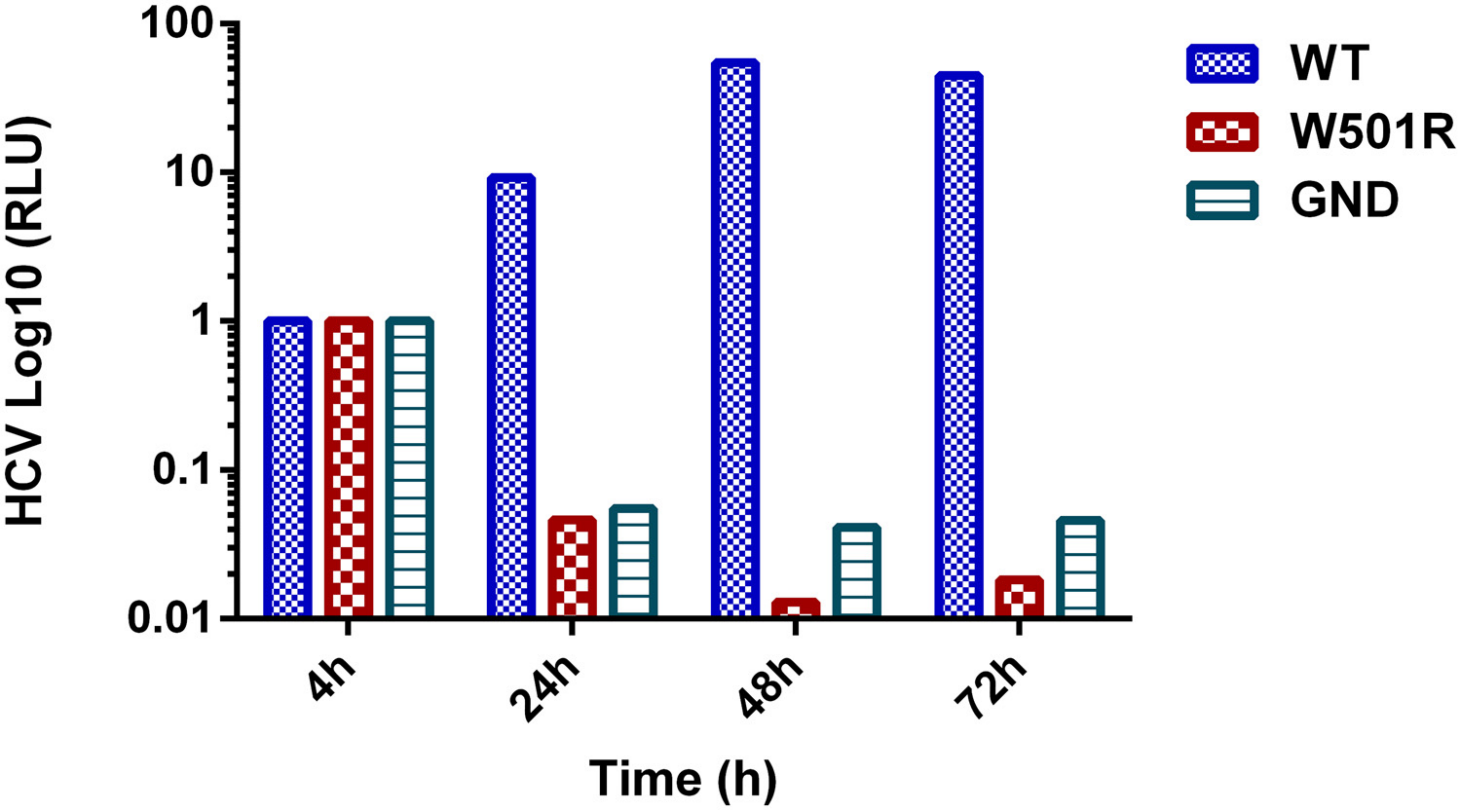
Transient subgenomic HCV replicon assays in duplicates. RLU—Relative Light Unit. The luciferase activity was measured in cell lysates at 4, 24, 48 and 72 h after transfection of RNA transcripts from pSGR-luc-JFH1 (WT), pSGR-Luc-W501R-JFH1 (W501R) and pSGR-Luc-JFH1/GND (GND). Data were normalized for luciferase activity values at 4 h. Bars represent the mean of two independent experiments with variability below 15%.

Lam & Frick [23] previously showed that a genotype 1b subgenomic HCV replicon does not tolerate a mutation encoding a W501A substitution in NS3, but the impact of a W501R mutation on subgenomic HCV replicons has not yet been reported. We therefore used a genotype 3a (S52) replicon and the more robust 2a (JFH1) replicon to examine the phenotype of the W501R allele. For the assays using the genotype 2a replicons, RNA transcribed from either pSGR-Luc-JFH1, pSGR-Luc-W501R-JFH1 or pSGR-Luc-JFH1/GND was used to transfect Huh-7.5 cells. Luciferase levels were then measured 4, 24, 48 and 72 h after transfection, and values obtained at 24, 48 and 72 h were normalized to values obtained at 4 h. The wild type Luc-JFH1 subgenomic replicon luciferase level increased from 4h to 48 h after transfection, and it remained the same thereafter. In contrast, the luciferase levels after transfection with either the Luc-W501R replicon or the Luc-GND replicon did not increase after transfection. Instead, they declined after transfection (Fig 5). Similar results were obtained with the genotype 3a replicons. Transcribed RNAs from pS52/SG-Feo (AII) and pS52/SG-W501R-Feo were used to electroporate Huh7.5 cells, which were selected with G418 for 15 days. After two weeks, only one colony was observed in the plate containing cells transfected with the W501R replicon, but cells transfected with wild-type RNA yielded 259 colonies (Fig A in S1 File).

## Discussion

In this study, we analyzed the impact of a W501R substitution on NS3 helicase isolated from HCV genotype 3a, and the phenotype of such an allele in HCV replicons. The work was performed to follow-up a prior study where Provazzi *et al*., identified the natural occurrence of the W501R allele in a Brazilian relapse patient infected with HCV genotype 3a [35]. The earlier work suggested that, unlike what has been observed with HCV genotype 1, HCV genotype 3a might tolerate such a mutation. For example, genotype 3a helicase might still be active without Trp501, or genotype 3a might be able to replicate without a functional helicase. Our data did not support either idea. Instead, our results suggest that this mutant allele remains circulating due to another mechanism. For example, the helicase function might be complemented in *trans* by wild type NS3 encoded by other quasi-species present in the patient.

Trp501 is one of the most widely studied NS3 residues, but all prior work was performed with enzymes isolated from HCV genotype 1 [29,47–50]. When we analyzed the substitution in a genotype 3a context, most results were similar to those reported earlier. For example, Kim *et al*. studied the W501R substitution in a genotype 1 background, and their results were similar to those reported here [29]. However, unlike other studies, we were able to detect some unwinding activity catalyzed by the W501R protein, although the activity was profoundly lower than that seen with wild type. We also showed that the W501R protein retains most of its nucleic acid binding capacity and ability to hydrolyze ATP in a nucleic acid stimulated manner. Numerous atomic structures of HCV NS3 helicase have revealed precisely how NS3 interacts with both ATP and nucleic acid [27,28,30,51–54]. NS3h structures solved in the presence of DNA reveal that Trp501 stacks against the 3’-terminal DNA base. This observation led Kim *et al*.[27] to propose that ATP binding, and the subsequent closure of the cleft between domains 1 and 2, leads to a ratcheting of Trp501 past 1 or 2 nucleotides allowing the protein to move on RNA like an inchworm [27]. Protein with the W501R substitution unwinds DNA and RNA much more slowly than wild type (Figs 1 & 2), but this defect is not due to a loss in DNA binding ability (Fig 3) or an ability to cleave ATP (Fig 4).

The effect of Trp501 substitution on HCV RNA replication was first studied by Lam & Frick [23], who showed that a genotype 1b replicon with a W501A mutation had no replication activity, while one with a W501F mutation did, showing that an aromatic ring at NS3 position 501 is essential for HCV RNA replication. In the present study, the W501R substitution was also introduced into genotype 2a and 3a subgenomic replicons. As was observed with genotpe 1b, severe defects in replication were observed.

The patient carrying the helicase W501R allele was a 46 year-old male genotype 3a HCV patient. He was treated with pegylated interferon alpha plus ribavirin for 24 weeks, and at the end of therapy HCV RNA was not detectable in his blood. However, three months after the end of the treatment, HCV RNA returned and was isolated from his serum samples. Only one of the 72 cDNA clones isolated from the patient’s serum encoded NS3 with an arginine at position 501. All other cDNA clones encoded a Trp at position 501. From this finding, and considering the gravity of the mutation, arose the need to investigate the W501R substitution in a genotype 3a background. We chose to study its effect on enzyme assays and replicon replication in cells. The data collected here confirm that the tryptophan at position 501 NS3 seems to be fundamental for genotype 3a HCV replication in cell culture, as was previously observed with genotype 1.

The HCV genome evolves rapidly because the NS5B RNA-dependent RNA polymerase lacks proofreading ability. As a result of the large amount (10^12^) of virions produced each day and the rate of incorrect nucleotide insertions, about 10^3^ to 10^4^ base substitutions occur each year at each site of the HCV genome during chronic infections [55,56]. Recombination also might enhance genetic diversity, given the fact that naturally occurring inter genotypic recombinant viruses have been identified [57–59]. As a result HCV circulates as closely heterogeneous related sequence variants in HCV patients [60–64]. The viral population typically consists of a dominant sequence, resulting from combining the most common nucleotide at each position of the genome, and of sequences differing from the dominant sequence to various extents [60–62,64,65]. Genetic heterogeneity in RNA viruses extends throughout their entire genome [66]. Sequence analysis of 18 full-length HCV genomes recovered from the serum of a patient acutely infected with HCV confirmed such heterogeneity [66]. Similar results were obtained from nine cloned full-length sequences obtained from acute-phase plasma of an experimentally infected chimpanzee [67]. Moreover, deletion mutants were detected in sera of HCV infected patients [68,69].

We are confident that the W501R allele was not selected as an artifact of the cloning procedures [70] for several reasons. First, molecular cloning of the RT-PCR products followed by sequencing of individual clones is the most accurate and reliable PCR-based technique [71]. Second, the original vector used for sequencing did not contain an *E*. *coli* promoter near the cloning site to minimize any toxic effects that HCV genes might have on *E*. *coli*. Foreign gene toxicity to *E*.*coli* would have led to low cloning efficiency [70], which was not observed in this project. Numerous different molecular clones were included in our analysis to obtain an adequate representation of the viral population and avoid an artifactual simplification of the sequences. Third, amplification of cDNAs was done with a high-fidelity DNA polymerase (see Methods), which minimizes the possibility that the mutation was produced by an artifactual result. In summary, we used a proof-reading polymerase to amplify cDNA from the patient and obtaining a significant number of clones was not an issue.

It remains a mystery why the W501R allele can survive in a patient if it is lethal even in a genotype 3a background. Based on the above observations, we suspect that the W501R allele might be complemented in *trans* to support its replication in this particular patient. We did not identify additional mutations in NS3 that could compensate the W501R substitution. The severe defect in replication observed in the replicon assay was produced from a HCV “mono” transfection without the other HCV variants that were also present in the patient, most of which lack the W501R allele. These other variants could support the replication by *trans*-complementation in chronically HCV infected patients, Recently, a mutant replicon containing the lethal NS3 W501A allele [23,29,49,72] was complemented in *trans* [73]. T*rans-*complementation was also previously reported using HCV harboring a mutation encoding a defective NS5A protein [74–76] and it was also described with the related *yellow fever virus* and *Kunjin virus* (KUN). In the flaviviruses, *trans-*complementation of NS1 defects occurs upon co-expression of recombinant, wild type NS1 with the mutant virus encoding defective NS1 [77,78]. In *Kunjin virus*, an Australian flavivirus closely related to West Nile virus, efficient *trans*-complementation was reported for mutations affecting the NS3 helicase and the NS5 RdRp [79]. The W501R allele, therefore, could be carried as a minor variant in chronically infected patients, and its replication could be attributed to *trans*-complementation of the mutant form of the HCV NS3 helicase by wild type variants. We intend to test this hypothesis in future studies.

In summary, this study confirmed the critical role of tryptophan at NS3 position 501 in HCV helicase-catalyzed nucleic acid unwinding, and we demonstrated that this role is critical for the replication of HCV genotypes 2a and 3a. We also demonstrated that this defect is not due to an inability to hydrolyze ATP or to bind to nucleic acids, and provide some evidence that such a lethal mutation might be complemented in *trans* in a clinical setting.

## Supporting Information

S1 File Effects of the NS3 W501R substitution on HCV helicase isolated from a Genotype 3a virus (Table A). Representative images of colony-forming assay for genotype 3a replicon cells assay (Fig A).
(PDF)
