# Supplementary material for: Analysis of the Enzymatic Activity of an NS3 Helicase Genotype 3a Variant Sequence Obtained from a Relapse Patient
Source: PLoS One. 2015 Dec 10;10(12):e0144638. doi: 10.1371/journal.pone.0144638 (PMC4684341; doi:10.1371/journal.pone.0144638)
Supplement: S1 File — Representative images of colony-forming assay for genotype 3a replicon cells assay (Fig A). (PDF) [file pone.0144638.s001.pdf]

Supporting Information

Table A: Effects of the NS3 W501R substitution on HCV helicase isolated from a Genotype 3a virus.

Assay	Parameter	Genotype 3a Wild type	Genotype 3a W501R	Genotype 1b Wild type
DNA Unwinding[a]	K_m (nM)	8.1± 3.1	320±120 (40)	24 ± 11
	V_{max} (kRFU/min)	17 ± 4	1 ± 0.2 (17)	7 ±1
RNA Unwinding[b]	K_m (nM)	21±9	22±20 (1)	n.d
	V_{max} (RFU/min)	2,200±700	25±7 (88)	n.d
DNA Binding[c]	K_d(nM)	2.5±0.3	4.5±0.8 (1.8)	5.2±0.4
Poly(U) Stim-ulatedATPas[d]	K_{act}(nM)	5.2±2.9	4.1±2.2 (1.3)	10±2
	k_{cat} (s^{-1})	52±4	46±3 (1.1)	24±1
Poly(A) Stim-ulatedATPase[d]	K_{act}(nM)	17±4	14±4 (1.2)	370±120
	k_{cat} (s^{-1})	43±2	33±2 (1.3)	23±2

[a]*Analysis of initial unwinding rates observed DNA unwinding assays with various concentrations of each protein (see Figure 1).*

[b] *Analysis of initial unwinding rates observed RNA unwinding assays with various concentrations of each protein (see Figure 2).*

[c] *Analysis of the relative amount of NS3h bound to an oligonucleotide at various concentrations of each protein (See Figure 3)*

[d] *Analysis of initial rates of NS3-catalyzed ATP hydrolysis observed with various RNA of each protein (see Figure 4).*

For each of the above assays, parameters calculated using non-linear regression analysis are shown with errors representing 95% confidence intervals in the curve fit. Fold change between the W501R allele and wildtype are shown in parentheses. n.d.=not determined.

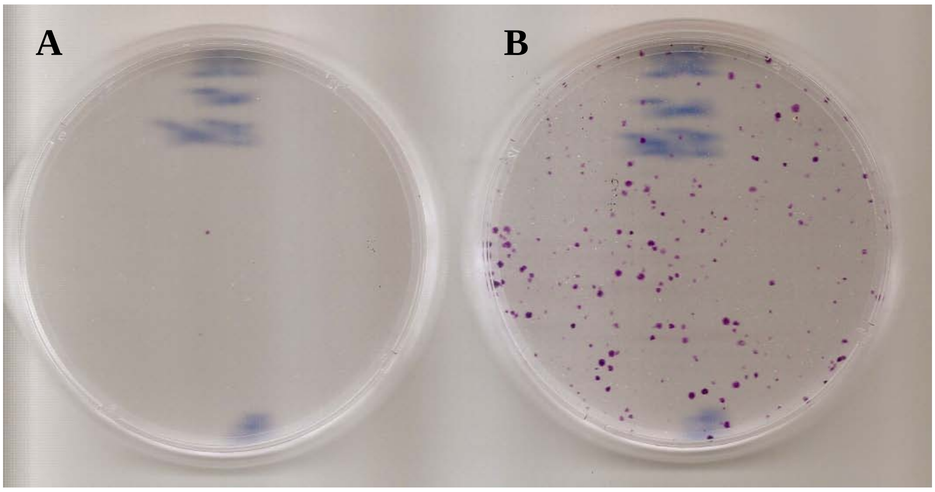

Figure A: **Representative images of colony-forming assay for genotype 3a replicon cells assay.** Transcribed RNAs from pS52/SG-Feo (AII) and pS52/SG-W501R-Feo were electroporated on Huh7.5 cells and treated with G418 for approximately 15 days. After two weeks only one colony was observed in the plate containing the cells electroporated with W501R mutation (A) mean while with the wild-type RNA for this mutation we observed in 259 colonies (B).
